# Genetic Characterization of a Core Set of a Tropical Maize Race Tuxpeño for Further Use in Maize Improvement

**DOI:** 10.1371/journal.pone.0032626

**Published:** 2012-03-07

**Authors:** Weiwei Wen, Jorge Franco, Victor H. Chavez-Tovar, Jianbing Yan, Suketoshi Taba

**Affiliations:** 1 National Key Laboratory of Crop Genetic Improvement, Huazhong Agricultural University, Wuhan, Hubei, China; 2 International Maize and Wheat Improvement Center (CIMMYT), El Batan, Mexico; 3 International Institute of Tropical Agriculture (IITA), Ibadan, Nigeria; University of Guelph, Canada

## Abstract

The tropical maize race Tuxpeño is a well-known race of Mexican dent germplasm which has greatly contributed to the development of tropical and subtropical maize gene pools. In order to investigate how it could be exploited in future maize improvement, a panel of maize germplasm accessions was assembled and characterized using genome-wide Single Nucleotide Polymorphism (SNP) markers. This panel included 321 core accessions of Tuxpeño race from the International Maize and Wheat Improvement Center (CIMMYT) germplasm bank collection, 94 CIMMYT maize lines (CMLs) and 54 U.S. Germplasm Enhancement of Maize (GEM) lines. The panel also included other diverse sources of reference germplasm: 14 U.S. maize landrace accessions, 4 temperate inbred lines from the U.S. and China, and 11 CIMMYT populations (a total of 498 entries with 795 plants). Clustering analyses (CA) based on Modified Rogers Distance (MRD) clearly partitioned all 498 entries into their corresponding groups. No sub clusters were observed within the Tuxpeño core set. Various breeding strategies for using the Tuxpeño core set, based on grouping of the studied germplasm and genetic distance among them, were discussed. In order to facilitate sampling diversity within the Tuxpeño core, a minicore subset of 64 Tuxpeño accessions (20% of its usual size) representing the diversity of the core set was developed, using an approach combining phenotypic and molecular data. Untapped diversity represents further use of the Tuxpeño landrace for maize improvement through the core and/or minicore subset available to the maize community.

## Introduction

Knowledge of genetic diversity within and among maize landraces is essential for effectively managing the conservation of landraces and using them in plant breeding. Maize landraces have genetic diversity in terms of plant and ear morphology, adaptation, and consumer traits such as grain quality and yields. Following studies based upon chromosomal knob morphology [1], [2] and isozyme markers [3]–[8], several analyses of maize landraces using DNA markers have been carried out [9]–[12]. Based on genotyping 193 landrace accessions at 99 microsatellite loci, Matsuoka et al. [9] presented phylogenetic analysis indicating a single domestication for maize and developed a scenario for its spread through the Americas. Reif et al. [10] used 25 simple sequence repeat (SSR) markers to characterize 25 maize race accessions from Mexico and examined their relationships on the basis of morphological data. Vigouroux et al. [11] analyzed the population genetic structure of maize races by genotyping 964 individual plants, representing most of the entire set of about 350 races native to the Americas, with 96 microsatellites. They identified the highland of Mexico and the Andes as potential sources of genetic diversity, which are currently underrepresented among elite lines in maize breeding programs. Most recently, Sharma et al. [12] revealed significant phenotypic and microsatellite-based genetic diversity in 48 landrace accessions in India, and identified promising accessions which could be utilized for introgression of novel traits in broad-based pools/populations.

The tropical maize race Tuxpeño has been incorporated in pools and populations in CIMMYT [13], where pools are maize populations with a broad genetic base. Its productivity *per se* and combining ability in crossing with race ETO developed at Estacion Tulio Ospina, Colombia is known as Tuxpeño-ETO heterotic patterns in tropical maize breeding [14]–[16]. It is predominantly a white dent with a cylindrical ear type. Some accessions of race Tuxpeño are yellow dent type, which were collected mainly in the Huasteca region of San Luis Potosi, Hidalgo, and Veracruz in Mexico. The long-term accessions evaluation experiments at CIMMYT planted 2,366 accessions of the race Tuxpeño since 1988. From them, 1,350 accessions were uniquely identified to be the race Tuxpeño. They are mostly from Mexico, but also include introductions from Brazil, Ecuador, Guatemala, and Venezuela. A multivariate cluster analysis of phenotypic data collected from seven trials was used to create a core set containing 321 accessions (23.7% of 1,350 Tuxpeño race accessions) of the race Tuxpeño [17]–[24].

CIMMYT has developed and released CIMMYT maize lines (CMLs) since 1984. The CMLs are carefully selected with good general combining ability (GCA) and a significant number of value-added traits such as drought tolerance, nitrogen use efficiency, acid soil tolerance, and resistance to disease and insect pests: (http://www.cimmyt.org/ru/component/content/article/459-international-maize-improvement-network-imin/434-cimmyt-maize-inbred-lines-cml). They are used as parental lines for the hybrids in one to several maize mega-environments (MEs). Two heterotic patterns were classified within CMLs (i.e. CML-A as dent kernel type and CML-B as flint kernel type). CMLs were developed from tropical, subtropical and highland white and yellow dent CIMMYT populations and pools, including germplasm from Central America, Caribbean, Mexico, South America, and USA. Some of them originated from populations and gene pools with a background of Tuxpeño germplasm.

The GEM project in the United States is designed to broaden U.S. maize breeding germplasm, representing a public-private sector collaboration in which elite tropical and sub-tropical germplasm (i.e. from non-Corn Belt dent races of maize) is crossed with private sector inbred lines (http://www.public.iastate.edu/~usda-gem/). GEM has used some of the elite germplasm of the Latin American Maize Project (LAMP) identified as a source of new genetic diversity for broadening the genetic base of U.S. maize hybrids, and breeding crosses are grouped into stiff stalk (SS) and non-stiff stalk (NSS) heterotic patterns [25]–[28]. As Tuxpeño germplasm has not been largely used in the GEM project, comparison of genetic diversity of them would be of interest to maize breeders.

In this study, the Tuxpeño core set containing 321 accessions, together with 14 U.S. landrace accessions, 11 CIMMYT populations, 4 temperate inbred lines, 94 CMLs and 54 GEM lines was characterized using SNPs across the maize genome. The objectives were to assess genetic diversity and genetic distance among the Tuxpeño core and other germplasm; to investigate potential utilization of the Tuxpeño core in maize improvement and to develop a minicore subset of the Tuxpeño core to facilitate sampling untapped alleles, if they existed.

## Materials and Methods

### Plant materials genotyped in this study

A total of 498 accessions were assembled in this study including 321 landrace accessions of Tuxpeño core set (two individual plants each accession except 24 accessions with one plant investigated), 94 CMLs, 54 GEM lines, 4 temperate maize inbred lines (Mo17, CI7_1, DAN340, K22_1), 14 landrace accessions from the U.S., and 11 CIMMYT populations (6 CIMMYT populations and 5 single cross hybrids between CMLs) (Table 1). Leaf samples of all 498 accessions (795 individual plants) were taken from individual plants at seedling stage. DNA was extracted using a modified CTAB procedure according to Murray et al. [29].

**Table 1:** Tuxpeño core and diverse germplasm used for genotyping.

Germplasm category	Origins of germplasm (country or state in Mexico)	Number of accessions
Tuxpeño core (landrace collection and populations)	Veracruz	74
	San Luis Potosi	57
	Chiapas	50
	Tamaulipas	23
	Guatemala	22
	Nayarit	20
	Sinaloa	11
	Hidalgo	7
	Jalisco	7
	Nuevo Leon	6
	Other states and countries	44
GEM recommended lines (SS/NSS)*	U.S.	54
CML: CIMMYT maize lines (A/B)*	CIMMYT	94
U.S. landraces (Southern and Corn Belt Dent)	U.S.	14
CIMMYT populations: breeding populations and single crosses	CIMMYT	11
Temperate inbreds	U.S.	3
	China	1
Total		498

* GEM SS: stiff stalk synthetic heterotic group; GEM NSS: non-stiff stalk synthetic heterotic group; CML-A: CIMMYT maize line of heterotic group A; CML-B: CIMMYT maize line of heterotic group B.

Within this Tuxpeño core, 295 accessions from Mexico, 22 accessions from Guatemala, 2 accessions from Brazil, and one each from Ecuador and Venezuela were included. Thus, the geographic origins of the Tuxpeño core are from Guatemala and Chiapas-Nuevo Leon (east coast), Veracruz-Nayarit (central region), and Colima-Sinaloa (west coast) of Mexico. The set of 94 CMLs includes lines from CIMMYT heterotic group A (n = 48) and B (n = 38), and 8 lines of A/B pattern. These lines were first chosen in seed production nurseries of CIMMYT maize germplasm bank for well adapted lines in the CIMMYT tropical and subtropical stations at Agua Fria and Tlaltizapán in cycle A, 2008. Thirty-five lines included in the U.S. GEM panel are stiff stalk (SS) heterotic pattern and 19 lines are non-stiff stalk (NSS) heterotic pattern. GEM SS lines included 25% germplasm of tropical hybrids from Brazil, Mexico, and Thailand, and landraces from Argentina, Brazil, and the Caribbean (Cuba), and 75% of elite temperate germplasm. GEM NSS lines included 25% germplasm of landraces from Brazil, Caribbean (Saint Croix), Chile, Mexico, Uruguay, and a hybrid of DKXL370 (Brazil). Within other germplasm, entries of “Across 8443”, CML 247 (G.24)×CML 254 (P.21), population 21, population 43, CML 444 (P.43), and CML 445 (Tux. Sequilla) have a background of mainly Tuxpeño germplasm. Pop. 28 and Pool 26 are yellow dent with slight Tuxpeño germplasm. Pop.32 (ETO Blanco) and Pop.23 (Blanco Cristalino: Pool 23) are white flint populations. Hybrids are included to represent those tolerant to drought, including lines with Tuxpeño background. CML395 (IITA 90323), CML 202 (ZSR923), CML312SR (P.500+SR), CML442 (Recycled in M37W/ZM607) have diverse origins. 14 U.S. landrace accessions are southern dent and Corn Belt dent. Detailed information of these lines collected and characterized in this study is listed in Table S1.

### Phenotypic evaluation and formation of Tuxpeño core set

Seven trial sets mentioned above were conducted during 1988 to 2008 at three CIMMYT experimental stations (i.e., Tlaltizapán, 18°41′48″N, 99°07′48″W, 940 m above sea level; Agua Fria, 20°27′00″N; 97° 38′ 24″W, 100 m above sea level; and Poza Rica, 20° 33′ 00″N; 97° 27′ 00″W, 60 m above sea level). The experimental design used alpha lattice with two replications. Each plot consisted of two 5 m rows with 75 cm apart between rows. Two seeds per hill were sown and later thinned to establish 32 plants per plot. Six check entries were included in each trial at each experiment station. Forty-four traits were evaluated for each accession, including morphological (plant height; ear height; ratio of ear height to plant height; tillering in scale; tassel type; percentage of erect plants; grain type; grain color), agronomic (days to 50% anthesis; days to 50% silking; ratio of anthesis to silking; foliar disease scale; root lodging (%); stalk lodging (%); number of plants harvested; number of ears harvested; ratio of harvested ears to harvested plants; field ear weight per plot (kg); rating on ear rot; rating on easiness of shelling; ear quality; grain moisture (%); grain shelling (%); adaptation in scale; agronomic scale; ratio of grain yield (kg) to grain moisture(%); yield per hectare (kg/ha)), vegetative (germination (%); rating on seedling vigor; number of leaves above the ear; days to leaf senescence; ratio of days to silking to days to leaf senescence; rating on forage production; rating on pubescence; rating on husk cover) and reproductive traits (ear length; ear diameter; kernel length; kernel width; kernel row number per ear; ratio of ear diameter to ear length; cob diameter; ratio of cob diameter to ear diameter; ratio of kernel width to kernel length). Detailed information of these traits can be found in Table S2. A multivariate cluster analysis (Ward-MLM) and a sample allocation strategy-D method and selection indexes (ESIM), were used to select core set to represent phenotypic diversity of the race Tuxpeño [17]–[23]. All trait data of discrete and continuous variables (44 traits in total) were included in calculating Gower distance among the accessions [24]. Based on the Gower distance, Ward was used to make a preliminary grouping, which was improved by MLM using maximum likelihood estimation. For each accession in the core set, the accession name, trial set in which they were evaluated, race classification, the value of each trait in the separate trial sets and the mega-environments (MEs) that they originated from are listed in Table S2.

### SNP genotyping

Genotyping was performed using Illumina GoldenGate assay on 1,536 bi-allelic SNP markers developed by Yan et al. [30]. The details of the SNP genotyping procedure and allele scoring have also been described [30]. The software Illumina BeadStation 500 G (Illumina, Inc., San Diego, CA, USA) was used for SNP genotyping according to the protocol described by Fan et al. [31]. Allele calling was re-checked manually and further analysis was carried out.

### Clustering analysis and genetic diversity

A neighbor-joining tree of these 498 entries was constructed based on the Modified Rogers genetic distance (MRD) using 1,041 SNPs. Briefly, pair-wise MRD between each two entries were calculated using an R (http://www.R-project.org) code, and neighbor-joining method implemented in the DARwin5 (http://darwin.cirad.fr/darwin) program was used on the matrix of distances to construct the dendrogram. An additional tree was constructed to show the relationship among different germplasm groups (Tuxpeño core, CML-A, CML-B, CML-A/B, GEM-SS, GEM-NSS, CIMMYT populations, U.S. landraces), based on the Nei's genetic distance [32]. Bootstrap support for this tree was determined by resampling across 1,041 SNP loci for 1000 times. The output of each bootstrap sample was summarized to obtain a consensus tree.

The genetic diversity parameters gene diversity and observed heterozygosity were quantified for sets of entries. Gene diversity, often referred to as expected heterozygosity, is defined as the probability that two randomly chosen alleles from the population are different. The estimator of gene diversity is defined for the r^th^ locus as 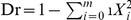, where m is the number of alleles and *X_i_* is the population frequency of the *i* ^th^ allele at locus *r* [33].

### Adaptation and genetic divergence of Tuxpeño core

A GIS–based approach for defining global maize production environments called “mega-environments (MEs)” has been useful for targeting maize germplasm for the introduction and adaptation trials [34]. The program DIVA-GIS (http://www.diva-gis.org/) was used to assign the maize growing environments based on the altitude, latitude and longitude information of the accessions. The MEs of 299 Tuxpeño accessions were defined based on their available geographic information.

Within the Tuxpeño core, 277 accessions were classified into 10 subgroups according to the 10 major geographic regions (i.e. Guatemala and 9 states in Mexico: Chiapas, Hidalgo, Jalisco, Nayarit, Nuevo Leon, Sinaloa, San Luis Potosi, Tamauripas, Veracruz) where they were collected from (Table 1), based on available passport data. The program Arlequin [35] was used to perform analysis of molecular variance (AMOVA; [35], [36]) and investigate the population differentiation among these 10 subgroups; and statistical significance of each variance component as well as pair-wise Fst was assessed based on 1000 permutations of the data.

### Minicore subset formation

Data of 44 phenotypic traits (i.e. 31 continuous, 11 categorical and two nominal variables; Table S2, [21]) and genotypic data (1,433 SNPs covering 10 chromosomes) from evaluation of 321 Tuxpeño accessions were used to develop a minicore subset with a sample size equal to 20% of the entire core set size (that is 64 accessions). Morphological Gower distance [24] and MRD [37] were calculated between every pair of the 321 accessions and then combined following the Gower principle of using the average of both the two distances weighted by the number of variables included in the distance calculations, where MRD accounted for more weight than morphological distance because of more SNP numbers than number of phenotypic traits (i.e., 1,433 vs. 44). The resulting matrix D of combined distances showed to be an Euclidean distance matrix as all the Eigen values from the similarity matrix *S = 1−D* were positive values, that is *S* was a positive definite matrix.

Because the evaluation of phenotypic data was conducted in seven different sets of trials, a sequential strategy was used to obtain the mini core subset. First we defined the number of accessions to be selected from each trial set according to the diversity of each trial set. That is, the number of accessions we selected is proportional to the average of distances between accessions within each trial set:
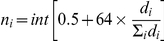
where *n_i_* is the number of accessions to be selected from the *i^th^* set, *d_i_* is the average of distances between accessions within the *i^th^* trial set, and 64 is the number of accessions to be selected to form the mini-core. Second, 1,000 mini-core subset candidates were randomly and independently drawn following a stratified random sample process of selection where each set was a stratum; then for each candidate subset the average distance between its 64 accessions was calculated. Finally, the candidate showing maximum average distance between accessions was selected to be the mini-core subset [38].

To evaluate the mini-core subset we used three concepts: (1) the increase of the average of distances between accessions in the mini-core in respect to the core set; (2) comparison of allele richness (expected and observed heterozygosity); (3) comparison of means, standard errors, and ranges between core and mini-core, and calculus of the range recuperation (RR, %) in the mini-core. As discussed by Marita et al. [39], allele richness is an evaluation from the point of view of taxonomists or geneticists looking for core subsets ensuring the inclusion of restricted or rare alleles; while distances between accessions is an evaluation from the point of view of breeders, looking for the inclusion of “generalized” alleles.

## Results

### Genotypic data

A total of 1,443 polymorphic SNPs (93.3%) were successfully called, with less than 10% missing data in 350 accessions (including 321 Tuxpeño core, 14 U.S. landraces, 11 CIMMYT populations and 4 temperate inbreds, 647 plants in total). They were evenly distributed across the whole maize genome, with coverage ranging from 103 SNPs on chromosome 10 to 213 SNPs on chromosome 1 (Table S3). Ninety-four CMLs and 54 GEM lines were genotyped with a set of SNPs [40] that has 1,041 markers in common with the 1,433 SNPs (Table S3). Marker names and physical positions of these 1,433 SNPs are listed in Table S3, where 1,041 out of 1,433 SNPs used for genotyping 148 GEM and CML lines were marked.

### Dendrogram of all entries

**Figure 1:**
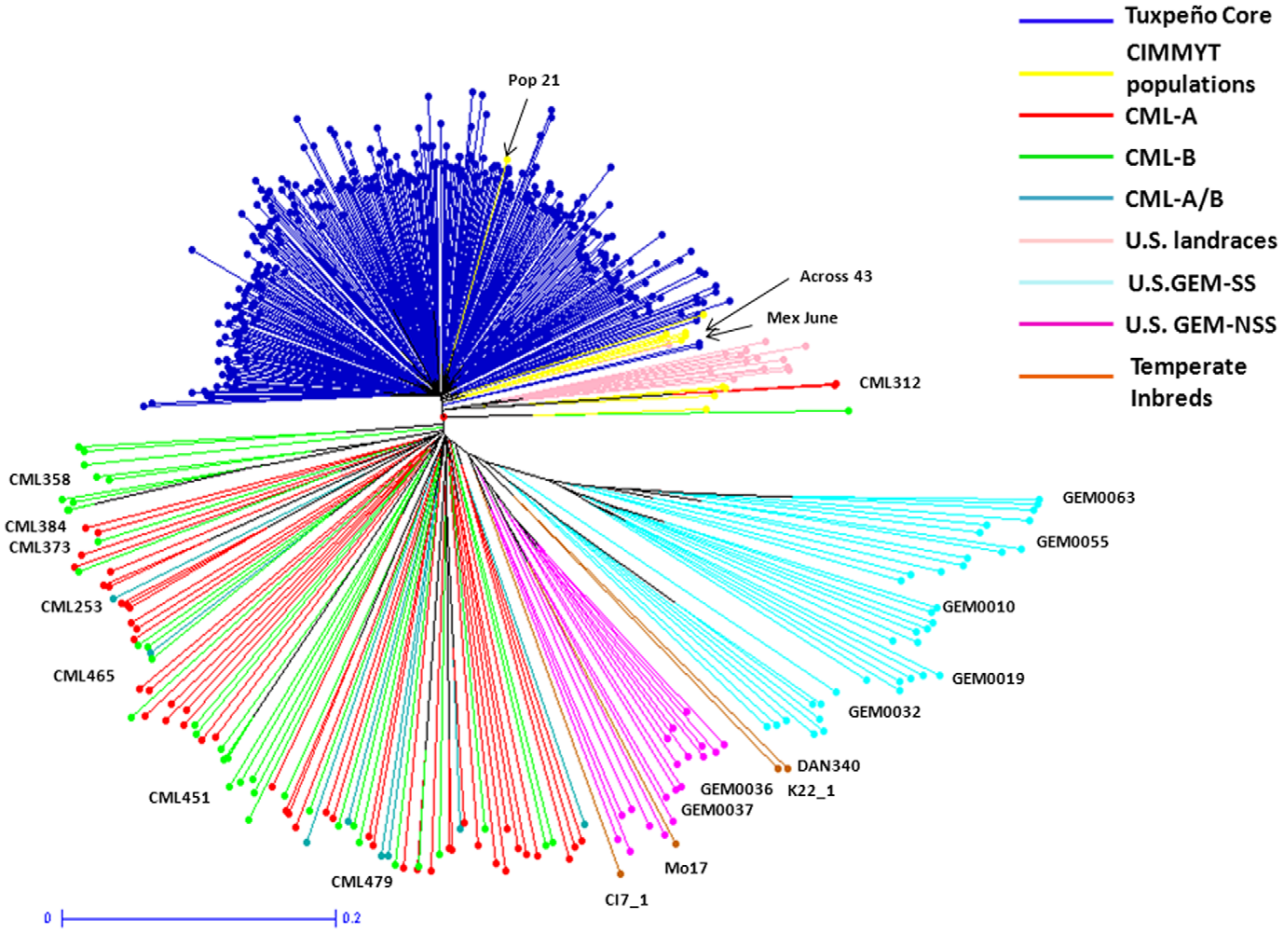
Neighbor-joining clustering of all 498 accessions based on the modified Rogers distance calculated using 1,041 SNPs.

**Figure 2:**
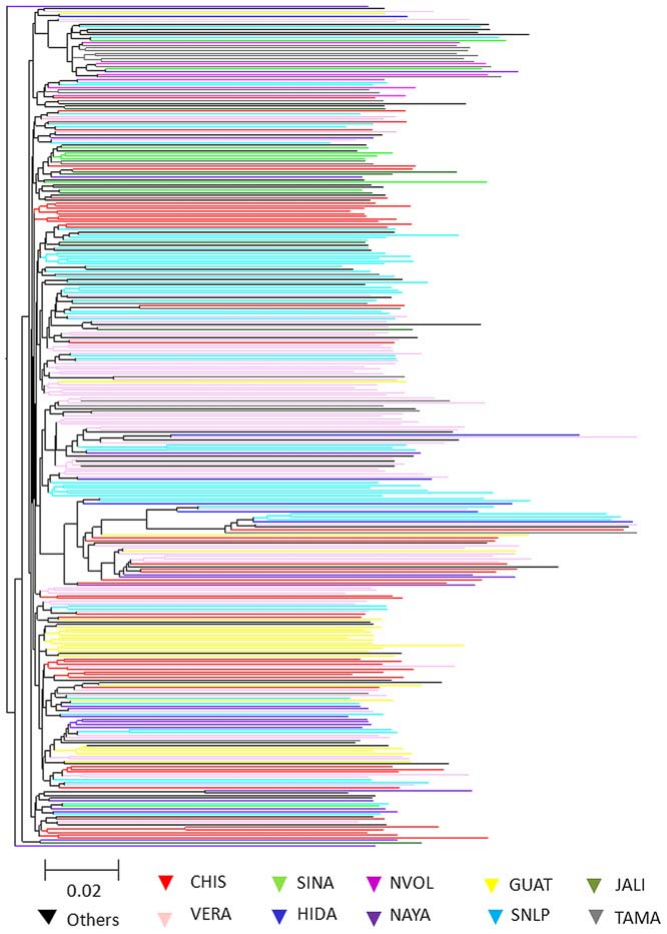
Neighbor-joining clustering of 321 Tuxpeño core based on the modified Rogers distance calculated using 1,041 SNPs.

The Neighbor-joining tree of all 498 entries is shown in Fig. 1, where lines from the same germplasm group (eg. Group of Tuxpeño core, CMLs and GEM lines) tended to clustered together. All U.S. landraces clustered together except one accession named “Mexican June”, which grouped with lines from CIMMYT populations (La Posta-Across 8443, Population 23, 28, 32, and Pool 24). Entries from CIMMYT populations were scattered next to the group of Tuxpeño core, except Population 21, which clustered amongst the Tuxpeño accessions. Pop 21 is composed of seven Tuxpeño race accessions and some families from Pool 24 (which is mainly based on Tuxpeño germplasm but includes also some materials from Central America). Lines from heterotic group SS and NSS of GEM were absolutely distinguished. Mo17 and the other three temperate inbred lines grouped with GEM lines; Mo17 and CI7_1 were clustered in the NSS group; K22_1 and DAN340 were clustered between NSS and SS group. However, lines from heterotic groups A and B of CMLs were not clearly separated. Grouping of different germplasm was also shown in Fig. S1, where bootstrap value (%) above 50% was shown. Tuxpeño accessions collected from the same region were not necessarily grouped together (Fig. 2).

### Genetic diversity among Tuxpeño core, GEM, CMLs and other germplasm

Gene diversity (expected heterozygosity) and observed heterozygosity of different sets of germplasm revealed by SNP markers are shown in Table 2. Using 1,433 SNPs, the set of U.S. landraces have higher values for gene diversity and heterozygosity than Tuxpeño core, temperate inbreds, and CIMMYT populations, which may be due to the inclusion of Southern dent and Corn Belt dent races in it [41]. The set of GEM lines has the highest values for gene diversity among all the germplasm assembled in this study, on the basis of 1,041 SNPs. This may result from the clear heterotic groups (SS and NSS) within GEM lines ([26];http://www.public.iastate.edu/~usda-gem/).

**Table 2:** Genetic diversity of Tuxpeño core and other diverse germplasms studied by two sets of SNP markers.

	Number of accessions	Number of plants	Gene diversity	Heterozygosity
Tuxpeño core	321	618	0.2926	0.2558
Tuxpeño mini core	64	121	0.2986	0.2623
U.S. landraces	14	14	0.3078	0.3610
CIMMYT populations	11	11	0.2667	0.2724
Temperate inbreds	4	4	0.2745	0.0551
Tuxpeño core	321	618	0.2997	0.2607
Tuxpeño mini core	64	121	0.3056	0.2671
U.S. maize races	14	14	0.3235	0.3792
CIMMYT populations	11	11	0.2735	0.2768
Temperate inbreds	4	4	0.2859	0.0464
CML	94	94	0.2990	0.0110
GEM	54	54	0.3891	0.1217

### Genetic distances among Tuxpeño core, GEM-SS, GEM-NSS, CML-A and CML-B

Pair-wise MRD among Tuxpeño core, CML heterotic groups A and B, GEM heterotic groups SS and NSS, as well as MRD within each group are shown in Table 3. According to Tukey-Kramer comparison of MRD means, larger genetic distances were observed between Tuxpeño core and GEM groups than that between Tuxpeño core and CML groups. MRD between CML heterotic groups A and B were less than that between GEM heterotic groups SS and NSS. The Tuxpeño core was closer to GEM-NSS group than GEM-SS group, according to the genetic distances. MRD within the Tuxpeño core was the least (Table 3). Relationship among different germplasm groups based on MRD was consistent with that based upon Nei's genetic distance, as revealed from Table 3 and Fig. S1.

**Table 3:** Average and standard error of modified Rogers pair-wise genetic distances studied by 1,041 SNP markers within (diagonal) and between (lower diagonal) Tuxpeño core (Tux.core), CML heterotic groups, and GEM heterotic groups; number of accessions per group (n); results of the Tukey-Kramer comparison of group means (lower letters).

Group	CML-A	CML-B	GEM-NSS	GEM-SS	Tux.core	n
CML-A	0.569±0.00082 e†					48
CML-B	0.570±0.00064 e	0.561±0.00104 f				38
GEM-NSS	0.585±0.00091 c	0.587±0.00102 c	0.502±0.00213 g			19
GEM-SS	0.657±0.00064 a	0.656±0.00072 a	0.636±0.00104 b	0.581±0.00112 d		35
Tux.core	0.480±0.00022 i	0.477±0.00025 j	0.490±0.00035 h	0.570±0.00026 e	0.335±0.00011 k	321

† Means followed by the same letter indicated no difference in the Tukey-Kramer test.

### Adaptation, genetic divergence and phenotypic variation of Tuxpeño core

The set of 321 Tuxpeño accessions represents 27 geographic regions (Mexican states and other countries) of the landrace adaptation, in which 10 major regions were identified. More than 5 accessions were collected from each of these 10 regions (Table 1). In total, 299 out of 321 accessions were classified into their corresponding MEs, based on available latitude, longitude and altitude data. A total of 171 accessions from 16 states of Mexico were classified as non-equatorial tropical/subtropical lowland wet mega-environment (day length: 12.5 to 13.4 hours, mean temperature ≥24°C, precipitation ≥600 mm and <2000 mm). The second largest group was classified into the tropical mid-altitude mesic mega-environment (day length: 11 to 12.5 hours, mean temperature >18°C and <24°C, precipitation ≥200 mm and <600 mm), in which 41 Tuxpeño core accession from Guatemala, and Chiapas, Tamaulipas, and Veracruz states in Mexico were collected. Twenty-six Tuxpeño core accessions were in non-equatorial tropical/subtropical lowland mesic (day length: 12.5 to 13.4 hours, mean temperature ≥24°C, precipitation ≥200 mm and <600 mm) and non-equatorial tropical/subtropical mid-altitude wet (day length: 12.5 to 13.4 hours, mean temperature >18°C and <24°C, precipitation ≥600 mm and <2000 mm) mega-environments, respectively, which are the third largest groups (Table S4).

The AMOVA (Table S5) revealed that a very low percentage (1.30%) of variation was partitioned among the 10 subgroups of Tuxpeño accessions. Only 9.74% of the variation was attributed to differences among individuals within these 10 subgroups. The majority of the variation was found within individuals (88.96%). Pair-wise Fst among these 10 subgroups showed that in general the accessions in Veracruz, Chiapas, and Guatemala were significantly differentiated from those in most of other states in Mexico (P≤0.01). Accessions from Hidalgo showed no significant differentiation as compared to those from all other subgroups (Table 4). However, genetic differentiation based on molecular data didn't completely concur with the morphological Gower distance (Table 5), suggesting no strong association between molecular and phenotypic data in this study. Most accessions in this Tuxpeño core are late white dent, with a few yellow late dent accessions collected from Huasteca regions of Veracruz, Hidalgo, and San Luis Potosi. CIMMYT populations have used most of them, but perhaps much less have been exploited from Chiapas and Guatemala.

**Table 4:** Pair-wise Fst studied based on 1433 SNPs for 10 subgroups of Tuxpeño core classified according to the regions they were collected from (i.e., 9 states of Mexico and Guatemala).

	CHIS	GUAT	HIDA	JALI	NAYA	NVOL	SINA	SNLP	TAMA	VERA
CHIS	0									
GUAT	0.015*	0								
HIDA	0.012	0.022	0							
JALI	0.014	0.029*	0.024	0						
NAYA	0.014*	0.029*	0.021	0.013	0					
NVOL	0.024*	0.040*	0.024	0.034	0.036	0				
SINA	0.013*	0.028*	0.018	0.016	0.014*	0.023	0			
SNLP	0.009*	0.022*	0.006	0.015	0.017*	0.018	0.011	0		
TAMA	0.015*	0.029*	0.013	0.024	0.024*	0.011	0.013	0.009*	0	
VERA	0.008*	0.013*	0.007	0.019*	0.018*	0.026*	0.017*	0.009*	0.014*	0

* Significant at the level P≤0.01.

CHIS = Chipas; GUAT = Guatemala; HIDA = Hildago; JALI = Jalisco; NAYA = Nayarit; NOVL = Nuevo Leon; SINA = Sinaloa; SNLP = San Luis Potosi; TAMA = Tamauripas; VERA = Veracruz.

**Table 5:** Average of Gower pair-wise phenotypic distances within (diagonal) and between (lower diagonal) 10 subgroups of Tuxpeño core originated from 9 states of Mexico and Guatemala; standard errors of the means (in parenthesis); results of the Tukey-Kramer comparison of means (lower letters); number of accessions in each subgroup (n).

	CHIS	GUAT	HIDA	JALI	NAYA	NVOL	SINA	SNLP	TAMA	VERA	n
CHIS	0.180 dc†										50
	(0.0019)										
GUAT	0.189 bdc	0.182 bdc									22
	(0.002)	(0.0044)									
HIDA	0.204 bac	0.212 bac	0.22 bac								7
	(0.0037)	(0.0057)	(0.0158)								
JALI	0.186 bdc	0.191 bdc	0.204 bac	0.199 bdac							7
	(0.0036)	(0.0055)	(0.01)	(0.0152)							
NAYA	0.212 bac	0.217 bac	0.203 bac	0.217 bac	0.201 bac						20
	(0.0022)	(0.0034)	(0.0059)	(0.0061)	(0.005)						
NVOL	0.205 bac	0.215 bac	0.198 bdac	0.211 bac	0.185 bdc	0.191 bdac					6
	(0.004)	(0.0062)	(0.0107)	(0.011)	(0.0061)	(0.0177)					
SINA	0.214 bac	0.224 a	0.207 bac	0.224 ba	0.197 bdac	0.182 bdc	0.199 bdac				11
	(0.003)	(0.0046)	(0.008)	(0.0083)	(0.0046)	(0.0082)	(0.0093)				
SNLP	0.195 bdac	0.203 bac	0.205 bac	0.205 bac	0.204 bac	0.194 bdac	0.203 bac	0.199 bac			57
	(0.0012)	(0.0019)	(0.0035)	(0.0035)	(0.002)	(0.0037)	(0.0028)	(0.0017)			
TAMA	0.169 dc	0.181 dc	0.186 bdc	0.178 dc	0.189 bdc	0.176 dc	0.188 bdc	0.176 dc	0.147 d		23
	(0.0019)	(0.0029)	(0.0053)	(0.0052)	(0.0031)	(0.0056)	(0.0042)	(0.0018)	(0.0038)		
VERA	0.211 bac	0.214 bac	0.211 bac	0.219 bac	0.200 bac	0.195 bdac	0.206 bac	0.206 bac	0.194 bdac	0.199 bdac	74
	(0.0011)	(0.0017)	(0.0031)	(0.0031)	(0.0018)	(0.0032)	(0.0024)	(0.001)	(0.0016)	(0.0013)	

† Means followed by the same letter indicated no difference in the Tukey-Kramer test.

The range and mean are summarized in Table 6 for certain important agronomical and yield-related or reproductive traits of the 321 Tuxpeño accessions evaluated in the seven trial sets. Wide variations were observed in days to 50% anthesis (AN), days to 50% silking (SI), plant height (PH), ear height (EH), ear length (EL) and ear diameter (ED). Other traits such as number of leaves above ear (LAE), kernel length (KL), kernel width (KWD), and ratio of kernel width to length (KWL) showed a relatively narrow range of variation.

**Table 6:** Statistical description of 14 agronomical and yield related traits of Tuxpeño core and selected mini-core evaluated from seven trials at CIMMYT stations.

	------- core (321) -------			----- mini-core (64) -----			
Trait†	Mean	Std Dev	Range	Mean	Std Dev	Range	RR§ %
AN (Day)	84.3	11.7	48.1	84.3	12.4	48.1	100.0
SI (Day)	86.6	12.0	54.1	86.6	13.0	54.1	100.0
PH (cm)	270.4	25.8	149.6	270.4	27.8	103.9	69.5
EH (cm)	172.7	23.9	143.4	172.7	27.6	122.1	85.1
LAE (No.)	6.3	0.5	2.3	6.3	0.5	2.3	99.2
EL (cm)	16.8	1.2	13.3	16.8	1.3	7.8	58.1
ED (cm)	4.7	0.3	3.2	4.7	0.3	1.7	53.1
KL (cm)	1.16	0.08	0.48	1.16	0.09	0.44	91.9
KWD (cm)	0.94	0.04	0.46	0.94	0.05	0.36	78.3
KRN (No.)	13.0	1.1	6.6	12.73	1.28	5.6	85.1
EDL (Ratio)	0.28	0.02	0.14	0.28	0.02	0.12	87.9
COB (cm)	2.42	0.31	3.17	2.42	0.30	1.42	44.7
CED (Ratio)	0.51	0.05	0.51	0.51	0.04	0.21	40.3
KWL (Ratio)	0.82	0.06	0.39	0.82	0.06	0.25	64.3

§ Percentage of the range in the entire core recovered by the minicore subset.

† AN = days to 50% anthesis; SI = days to 50% silking; PH = plant height; EH = ear height; LAE = number of leaves above the ear; EL = ear length; ED = ear diameter; KL = kernel length; KWD = kernel width; KRN = kernel row number; EDL = ratio of ear diameter to ear length; COB = cob diameter; CED = ratio of cob diameter to ear diameter; KWL = ratio of kernel width to kernel length.

### Minicore subset of Tuxpeño

A minicore subset containing 64 accessions was defined. The genetic diversity represented by gene diversity, heterozygosity and Gower distance (Gd) in the minicore and core collections were compared. Gene diversity and heterozygosity of the minicore subset were higher than those of the core set (Table 2). In addition, Gd of the minicore subset (0.3289) was higher than that of the core set (0.3159) as well. Finally the means, standard deviations and ranges of 14 agronomical and yield related continuous variables characterized for the entire core set were recovered in the minicore (Table 6). Thus, the minicore subset reduced the number of genotypes while maintaining the diversity of the core collection (i.e. reducing the presence of some redundancies in the entire core set), which is satisfactory. The collecting sites (states or departments in Mexico and Guatemala) and CIMMYT accession identification numbers (Acc.ID) of these 64 Tuxpeño minicore accessions are shown in Table S6.

## Discussion

### Genetic diversity of Tuxpeño core set and minicore subset

The Tuxpeño core set for breeding use was chosen to best represent phenotypic diversity within the race. They covered 23 States of Mexico, and parts of Brazil, Ecuador, Guatemala, and Venezuela, including landraces and old breeding populations. A relatively high gene diversity and heterogygosity were observed as revealed by SNP markers. In addition, the geographic locations (mega-environments) where the Tuxpeño core accessions were collected show a wide climatic range. This confirmed a previous study which indicated that Tuxpeño is the most widely adapted Mexican landrace, as it is found in 19 climatic types [42]. Environmental differences seem to drive the overall patterns of maize diversity [42], [43]. Ecogeographical information where the collections originated from is central to understanding the variety of other sites in which they can adapt to. Breeders can select the promising accessions with potential adaptation and use them in the breeding program. The minicore subset, as indicated from the present result, can capture the genetic variation present in the Tuxpeño core set. We used a strategy combining phenotypic and genotypic data to develop the minicore. A distance was defined using both phenotypic and genotypic variables to achieve effective classification of genotypes. Inclusion of morphological traits to measure the distance is better than using only genotypic or marker data, since they provided additional information generally independent of the genotypic information. The use of the weighted average of both morphological and genetic distance followed the Gower principle, in which more variables produce larger effects. Evaluation of agronomically important and stress-tolerant traits can be carried out using the minicore. Mining new alleles for useful traits either in the minicore or in the core is cost-effective, as the number of accessions is substantially reduced compared to that of the entire Tuxpeño race collection at the CIMMYT maize germplasm bank.

The present study on the core set of the largest collection in CIMMYT (i.e. race Tuxpeño) can be extended and applied to other landrace collections. As shown in Figure 2, relationship among the accessions does not necessarily follow the geographic pattern for the collection of the accessions. Hence, genotyping a large number of accessions and plants per accession would be necessary in order to establish relationship among the landraces and devise sampling strategy in the future.

### Grouping of Germplasm

Clustering analysis based on MRD and Nei's genetic distance revealed clear separation among different germplasm (Fig. 1; Fig. S1). No subclusters were formed within the Tuxpeño core, which is consistent with a high within individual variation (89%) revealed by AMOVA (Fig. 2, Table S5). A total of 94 CMLs were not well separated into A (mostly dent type) or B (flint type) patterns, as conventional heterotic groups classified by the CIMMYT breeders. This is as expected because most germplasm sources used to extract the lines were established based on a mixture of different racial complexes [44], [45]. Similar results were demonstrated in previous studies [46], [47]. For CMLs analyzed in this study, more than 50% of their base populations included Tuxpeño germplasm (dent kernel) in their formation as CIMMYT gene pools and populations used Tuxpeño germplasm for its high productivity *per se* and good combination with other germplasm (Table S1; [13]). This can be reflected by the relatively low genetic distance between the CMLs and Tuxpeño core (Table 3).

On the other hand, 54 U.S. GEM recommended lines showed two clear groups of NSS and SS heterotic patterns. The Tuxpeño core had the largest genetic distance from GEM-SS lines among its genetic distances from all other groups. In this study, larger genetic distance between tropical germplasm (i.e. Tuxpeño core, CML-A and CML-B) and SS were observed than that between tropical germplasm and NSS, which is consistent with a previous study [48]. A large genetic distance between heterotic germplasm can be useful for developing lines with good combining ability in hybrid breeding [49], [50]. GEM-SS can be an excellent heterotic germplasm against CML-A, CML-B and Tuxpeño germplsms, considering these CMLs analyzed in this study did not show large MRD from the other germplasm groups.

The gene diversity parameter used for evaluating the genetic diversity in this study is less sensitive to the sample sizes of the subsets [11], [51]. However, the allele number of each locus is restricted to a maximum of two when using bi-allelic SNP markers, which may cause limitations in genetic diversity measurement. Detection of genetic diversity with a large number of SNPs could mitigate the shortage. In addition, ascertainment biases might affect the measurement of diversity and population differentiation due to the use of SNP genotyping chips. The frequency of alleles may be affected and difference among temperate lines may be overestimated compared to that within tropical lines, because most SNPs (1106 out of 1536) used in the present study were developed from sequencing the set of 27 parental lines of the nested association mapping (NAM) population (i.e., SNPs were selected to maximize polymorphisms between B73 and 26 other inbred parental genotypes. About half of the 26 lines are tropical.) [30]. With the availability of maize genome and the advance of genotyping by sequencing technology, larger amount SNPs with good quality can be used for molecular characterization of maize landraces, which is possible to control ascertainment bias [52], [53], [54].

### Further use of Tuxpeño core set in maize breeding programs

Tuxpeño germplasm has been exploited in tropical maize improvement for its yield potential [55]–[57], superior plant type [58], [59], and resistance to drought and pests [60], [61]. They constitute the largest collection in the CIMMYT maize germplasm bank. Despite much larger genetic distances and allelic frequency differences between Tuxpeño and GEM groups than that between Tuxpeño and CML groups, the results of cluster analysis showed clear separation of CMLs from Tuxpeño. The divergence between them implies that there may be untapped allelic variations in Tuxpeño germplasm, which can be used for broadening the genetic diversity within CML-A or B groups.

The 54 GEM lines investigated in our study have a 50% or 75% background of temperate germplasm and a 25% or 50% background of tropical germplasm. The genetic diversity of GEM was broader in this study, compared to the tropical germplasm (i.e. CML and Tuxpeño). However, large allelic frequency differences between GEM and tropical germplasm imply that the tropical germplasm can be used in a temperate breeding program. Incorporation of elite tropical and subtropical germplasm into elite temperate germplasm to combine favorable alleles into germplasm pools adapted to temperate environments as well as to broaden its genetic base have been carried out in previous studies [62], [63]. Whitehead et al. [62] suggested that 25% elite exotic germplasm can be incorporated in the important U.S. heterotic groups without disrupting the highly productive combining ability for grain yield expressed in BSSS and non-BSSS hybrid combinations. On the other hand, GEM germplasm can be considered as an exotic source for improving tropical maize lines and populations. Promising results were observed in the breeding crosses, where clearer separation was observed between the F_1_ crosses from CML A×GEM-SS and CML B×GEM-NSS [40].

Larger separation between GEM heterotic groups (i.e. SS and NSS), compared to the genetic divergence between CML heterotic groups (i.e. CML-A and CML-B) provide tropical and temperate maize breeders with potential germplasm sources for hybrid maize breeding, in which the genetic distances between opposite heterotic lines and populations can be increased. For example, we can make allied breeding cross combinations between GEM-SS and CML-A (or Tuxpeño minicore), and between GEM-NSS and CML-B (or Tuxpeño minicore). GEM lines are subtropical-temperate adapted and more tropical germplasm should be incorporated for its use in tropical breeding. In the above breeding cross combinations, selection for tropically adapted SS-A heterotic pattern and NSS-B heterotic pattern is recommended for tropical maize breeding. Although Tuxpeño is one of the heterotic patterns in tropical maize breeding, it may contribute to enhancing GEM-SS heterotic lines. The same can be done with Tuxpeño minicore for enhancing CML-A and CML A/B in the similar grain types. Selection for adaptation and increasing genetic divergence must be done as a priority using standard breeding procedures. As a result, superior lines and hybrids can be developed in the adapted regions.

In addition, short stature improved populations and lines of Tuxpeño germplasm are good sources for improving the farmers' landraces, without altering grain type and adaptation. CIMMYT maize genebank has used the improved gene pools and lines in participatory maize breeding in the state of Oaxaca, Mexico (Taba et al. unpublished data; [20]) for evolutional maize germplasm conservation. In this way, genetic diversity of the race can be maintained *in situ* on farm [64] and modern maize production can be realized with small scale farmers.

## Supporting Information

Figure S1 Dendrogram of different germplasm groups (Tuxpeno core, CML-A, CML-B, CML-A/B, GEM-SS, GEM-NSS, CIMMYT populations, U.S. landraces). Clades with greater than 50% bootstrap support are indicated.
(PPT)

Table S1 Information of lines collected and characterized in this study.
(XLS)

Table S2 Detailed information of 321 Tuxpeño accessions.
(XLS)

Table S3 Information of 1,433 SNPs used in this study.
(XLS)

Table S4 Distribution of Tuxpeño core accessions (299 accessions with available information) by the collection information in maize mega-environments (MEs) defined by a GIS approach.
(DOC)

Table S5 Analysis of molecular variance of 10 subgroups of Tuxpeño accessions classified according to the 10 major geographic regions where they were collected.
(DOC)

Table S6 Collecting sites (states or departments in Mexico and Guatemala) and CIMMYT accession identification number (Acc.ID) of 64 Tuxpeño minicore accessions.
(DOC)
