# Supplementary figures and images for: Genetic Characterization of a Core Set of a Tropical Maize Race Tuxpeño for Further Use in Maize Improvement
Source: PLoS One. 2012 Mar 7;7(3):e32626. doi: 10.1371/journal.pone.0032626 (PMC3296726; doi:10.1371/journal.pone.0032626)

## Slide 1
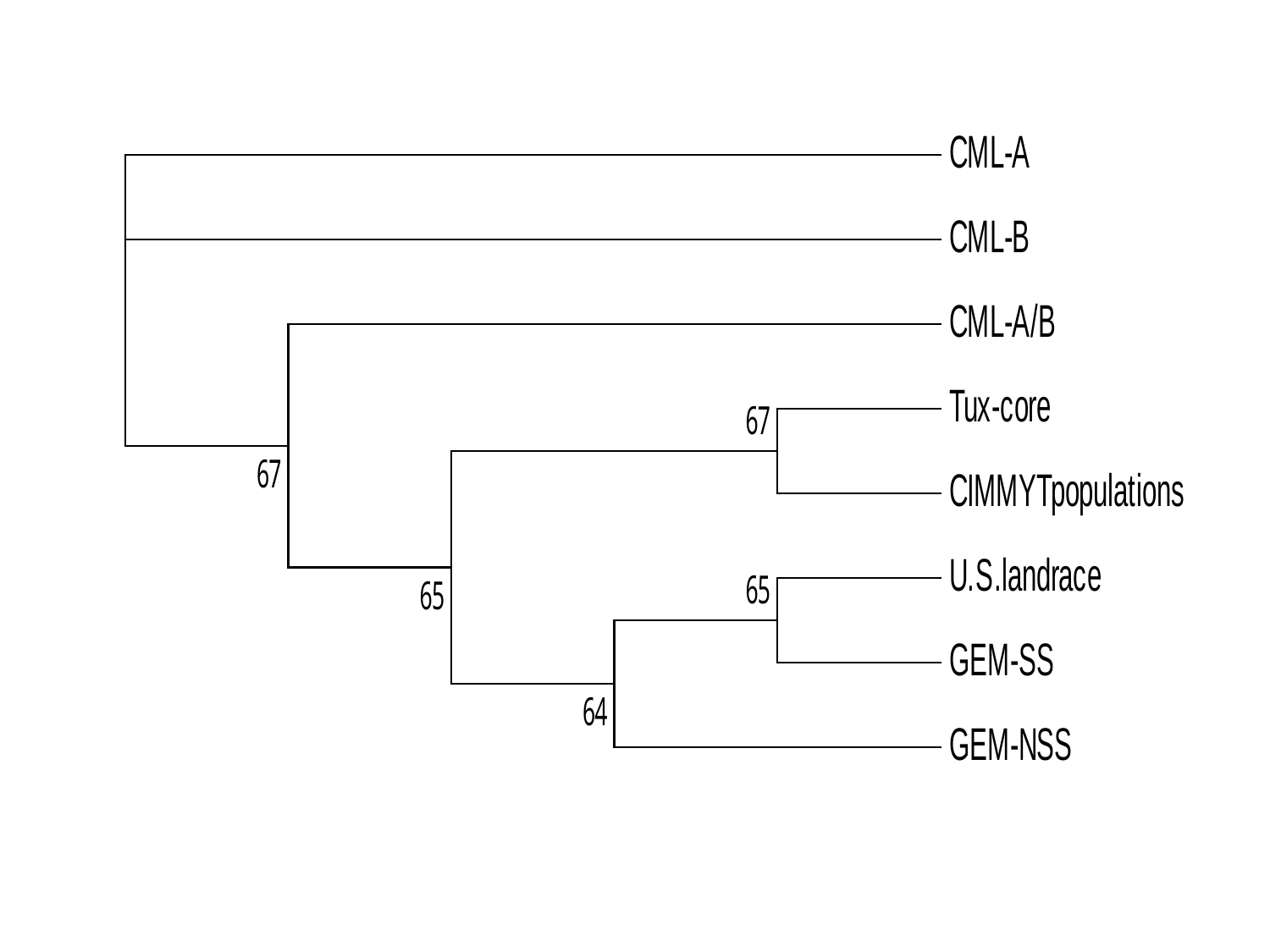

Supplement: Figure S1 — Dendrogram of different germplasm groups (Tuxpeno core, CML-A, CML-B, CML-A/B, GEM-SS, GEM-NSS, CIMMYT populations, U.S. landraces). Clades with greater than 50% bootstrap support are indicated. (PPT) [file pone.0032626.s001.ppt]
