# Supplementary material for: Genetic Characterization of a Core Set of a Tropical Maize Race Tuxpeño for Further Use in Maize Improvement
Source: PLoS One. 2012 Mar 7;7(3):e32626. doi: 10.1371/journal.pone.0032626 (PMC3296726; doi:10.1371/journal.pone.0032626)
Supplement: Table S4 — Distribution of Tuxpeño core accessions (299 accessions with available information) by the collection information in maize mega-environments (MEs) defined by a GIS approach. (DOC) [file pone.0032626.s005.doc]

Table S4. Distribution of Tuxpeño core accessions (299 accessions with available information) by the collection information in maize mega-environments (MEs) defined by a GIS approach.

| ME Classification | No. of accessions | Collection sites |
| --- | --- | --- |
| Non equatorial tropical/subtropical highland mesic | 3 | PUEB |
| Non equatorial tropical/subtropical highland wet | 3 | PUEB,GUAT |
| Non equatorial tropical/subtropical lowland mesic | 26 | SNLP,VERA,SINA,SONO,CAMP |
| Non equatorial tropical/subtropical lowland wet | 171 | MICH,OAXA,CHIH,TAMA,SNLP,VERA,  HIDA,JALI,NAYA,DURA,CHIS,QROO,  QUER,SINA,SONO,MORE |
| Non equatorial tropical/subtropical mid altitude mesic | 15 | CHIH,QUER,SNLP,HIDA,DURA,TAMA |
| Non equatorial tropical/subtropical mid altitude wet | 26 | CHIS,SNLP,GUAT,VERA,JALI,MORE,MICH |
| Temp/subtropical hot mesic | 1 | TAMA |
| Temp/subtropical hot wet | 1 | SINA |
| Too dry tropical mid altitude | 7 | SNLP,NVOL,COAH |
| Too dry tropical highland | 2 | COAH,SNLP |
| Tropical lowland mesic | 2 | TAMA,TABA |
| Tropical midaltitude mesic | 41 | GUAT,CHIS,TAMA,NOVL,VERA |
| Tropical midaltitude wet | 1 | GUAT |

CAMP=Campeche; CHIS=Chipas; CHAH=Chihuahua; COAH=Coahuila; DURA=Durango, GUAT=Guatemala; HIDA=Hildago; JALI=Jalisco; MICH=Michóacan; MORE=Morelo; NAYA=Nayarit; NOVL=Nuevo Leon; OAXA=Oaxaca; PUEB=Puebla; QROO=Quintana Roo, SINA=Sinaloa; SNLP=San Luis Potosi; SONO=Sonola; TABA=Tabasco; TAMA= Tamauripas; VERA=Veracruz.
