# Supplementary material for: Genetic Characterization of a Core Set of a Tropical Maize Race Tuxpeño for Further Use in Maize Improvement
Source: PLoS One. 2012 Mar 7;7(3):e32626. doi: 10.1371/journal.pone.0032626 (PMC3296726; doi:10.1371/journal.pone.0032626)
Supplement: Table S5 — Analysis of molecular variance of 10 subgroups of Tuxpeño accessions classified according to the 10 major geographic regions where they were collected. (DOC) [file pone.0032626.s006.doc]

Table S5. Analysis of molecular variance of 10 subgroups of Tuxpeño accessions classified according to the 10 major geographic regions where they were collected.

| Source of Variation | d.f. | Sum of squares | Variance components | Percentage of variation | *P*-value |
| --- | --- | --- | --- | --- | --- |
| Among subgroups | 9 | 4089 | 2.48 | 1.3 | <0.001 |
| Among individuals within subgroups | 525 | 109006 | 18.65 | 9.7 | <0.001 |
| Within individuals | 535 | 91125 | 170.33 | 89.0 | <0.001 |
| Total | 1069 | 204221 | 191.47 |  |  |
