# Supplementary material for: Genetic Characterization of a Core Set of a Tropical Maize Race Tuxpeño for Further Use in Maize Improvement
Source: PLoS One. 2012 Mar 7;7(3):e32626. doi: 10.1371/journal.pone.0032626 (PMC3296726; doi:10.1371/journal.pone.0032626)
Supplement: Table S6 — Collecting sites (states or departments in Mexico and Guatemala) and CIMMYT accession identification number (Acc.ID) of 64 Tuxpeño minicore accessions. (DOC) [file pone.0032626.s007.doc]

Table S6. Collecting sites (states or departments in Mexico and Guatemala) and CIMMYT accession identification number (Acc.ID) of 64 Tuxpeño minicore accessions.

| Region | Number of accessions | Accession ID number in CIMMYT collection |
| --- | --- | --- |
| Chiapas | 10 | 16286;18837;23164;23203;23209;24289;24297;25780;25794;25832 |
| Chihuahua | 1 | 13339 |
| Guatemala* | 8 | 27554;27601;27602;27603;27648;27658;27663;27666 |
| Hidalgo | 3 | 1928;29382;29384 |
| Morelos | 2 | 16311;23723 |
| Nayarit | 3 | 7094;7095;7100 |
| Oaxaca | 1 | 120 |
| Puebla | 2 | 1589;23666 |
| San Luis Potosi | 12 | 425;18567;18907;23677;25255;29369;29418;29421;29485;29489;29495;29498 |
| Population from Mexico | 3 | 5682;1233;4022 |
| Tamaulipas | 8 | 415;2545;17933;18920;24023;24751;24957;25806 |
| Population from Brazil | 1 | 14182 |
| Venezuela | 1 | 11175 |
| Veracruz | 9 | 697;702;3251;17954;20412;23244;23246;25078;25093 |

*includes departments of Jutiapa, Retalhuleu, Huehuetenango, and Peten.
